# Supplementary material for: The causal relationship between the human gut microbiota and pyogenic arthritis: a Mendelian randomization study
Source: Front Cell Infect Microbiol. 2024 Nov 26;14:1452480. doi: 10.3389/fcimb.2024.1452480 (PMC11629706; doi:10.3389/fcimb.2024.1452480)
Supplement: Supplementary file 1 [file DataSheet1.zip › mendelian test/ebi-a-GCST90027612.csv_leave_one_out.pdf]

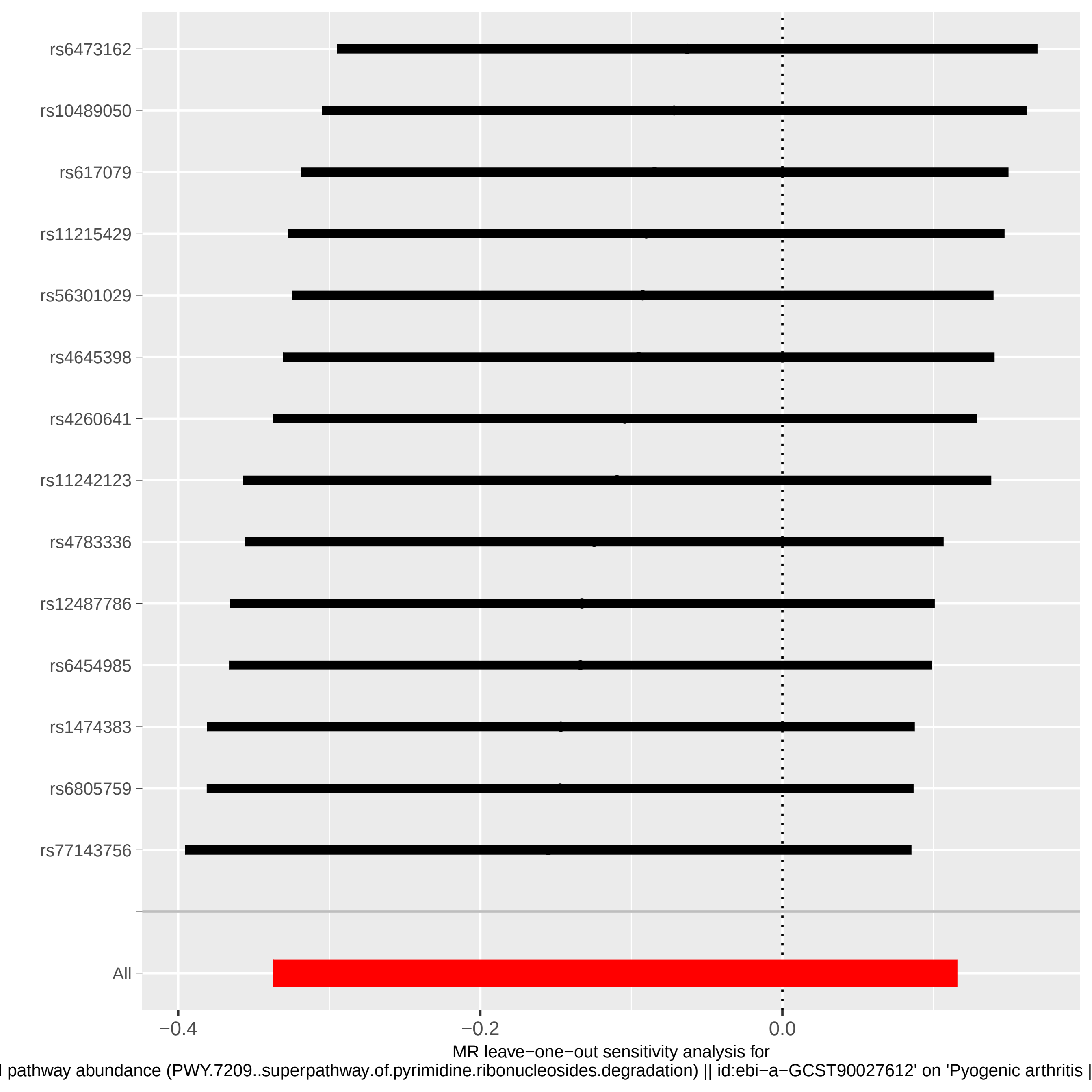

rs6473162
rs10489050
rs617079
rs11215429
rs56301029
rs4645398
rs4260641
rs11242123
rs4783336
rs12487786
rs6454985
rs1474383
rs6805759
rs77143756
All
−0.4
−0.2
0.0
MR leave−one−out sensitivity analysis for
l pathway abundance (PWY.7209..superpathway.of.pyrimidine.ribonucleosides.degradation) || id:ebi−a−GCST90027612' on 'Pyogenic arthritis |
